# Are We All Fools?

**Published:** 1898-07

**Authors:** 


### Are We All Fools?

The man who will invent some kind of a process, or a serum, that will actually destroy the notion that "there is always room at the top," should receive a position of $50,000 a year for the next 50,000 years! Twenty-five thousand students in our medical colleges last year! Think of it. Nothing in the world to make such a vast army, only because each thinks himself able to get where there is always room. There are hundreds of men who would be recognized as skillful as Senn in the use of the knife—if they had the opportunity. Do not think, not even for one little minute, that Dr. Polk, of New York, takes in $100,000 a year because he is just five times as talented as the man who takes in $20,000. If these 25,000 medical students thought they would have to fight their way along, struggle through immense difficulties, that many of them would surely fall by the way and that this "room-at-the-top" business is poetical fancy; we say, if this were better understood there would be the greatest outpouring from college halls that the world has ever seen. But fortunately for these schools, "ignorance is bliss, sure enough."—*Journal of Practical Medicine.*

